# Complete Genome Sequence of Chesapeake Bay Winter *Synechococcus* sp. Strain CBW1107, a Member of Subalpine Cluster II

**DOI:** 10.1128/MRA.01399-20

**Published:** 2021-02-18

**Authors:** Daniel Fucich, Yongle Xu, Ana Sosa, Rui Zhang, Nianzhi Jiao, Feng Chen

**Affiliations:** a Institute of Marine and Environmental Technology, University of Maryland Center for Environmental Science, Baltimore, Maryland, USA; b Institute of Marine Science and Technology, Shandong University, Qingdao, China; c State Key Laboratory of Marine Environmental Science, Fujian Key Laboratory of Marine Carbon Sequestration, College of Ocean and Earth Sciences, Xiamen University, Xiamen, China; d Southern Marine Science and Engineering Guangdong Laboratory (Zhuhai), Zhuhai, China; Georgia Institute of Technology

## Abstract

Here, we report the complete genome sequence of psychrotolerant *Synechococcus* sp. strain CBW1107, which was isolated from the Chesapeake Bay in winter. CBW1107 is a member of picocyanobacterial subalpine cluster II and exhibits greater cold tolerance than do most coastal and marine *Synechococcus* strains.

## Abstract (precis)

Here, we report the complete genome sequence of psychrotolerant *Synechococcus* sp. strain CBW1107, which was isolated from the Chesapeake Bay in winter. CBW1107 is a member of picocyanobacterial subalpine cluster II and exhibits greater cold tolerance, compared to most coastal and marine *Synechococcus* strains.

## ANNOUNCEMENT

The Chesapeake Bay (CB) is a large temperate estuary with strong seasonal variation of water temperature (0 to 28°C). The picocyanobacterial abundance in the CB varies from a few hundred cells per ml in winter to over 1 million cells per ml in summer (1). Distinct picocyanobacterial populations in winter and summer were found in the CB (2). Seventeen strains of *Synechococcus* were isolated from the Baltimore Inner Harbor in winter (3). These winter isolates are able to grow at low temperatures (4°C and 10°C) at which many coastal and open-ocean *Synechococcus* strains cannot survive. *Synechococcus* sp. strain CBW1107 is one of five CB winter isolates clustered in subalpine cluster II, which contains picocyanobacteria isolated from subalpine lakes, the Arctic Ocean, the Baltic Sea, and Long Island Sound (3).

CBW1107 was grown in SN15 medium (3). For DNA extraction, 20 ml of an exponential-phase culture (optical density at 750 nm [OD_750_], 0.8) was centrifuged at 10,000 × *g* for 10 min. Cell pellets were ground with liquid nitrogen, transferred into 2-ml tubes with cetyltrimethylammonium bromide (CTAB) lysis buffer (4), and then incubated for 60 min at 65°C in a water bath. Genomic DNA was then extracted by using the phenol-chloroform method described by Kan et al. (5). The complete genome sequence of CBW1107 was obtained using a combination of Illumina HiSeq and PacBio Sequel platforms at the Beijing Genomics Institute (BGI) (Shenzhen, China). The details of the sequencing reads can be found in Table 1. Default parameters were used for all software unless otherwise noted for the sequence processing and genome analyses. Four single-molecule real-time (SMRT) cell zero-mode waveguide arrays for sequencing were used with the PacBio platform to generate the subread set. The raw reads from the Illumina sequencing with an average 2×150-bp paired-end sequencing kit with low quality (score of ≤20) or high N nucleotide percentage (>10%), adapter reads, and duplicate reads were removed to obtain clean reads by using SOAPnuke version 1.5.6. For PacBio raw sequences, adapters and poor-quality reads were cut from polymerase reads to generate multiple subreads. Subreads with less than 1,000 nucleotides were filtered out, and the remaining subreads were integrated into one circular consensus sequencing (CCS) read of insert through the use of the following software: subreads were corrected using multiple programs (Pbdagcon, FALCON Consensus, and Proovread version 2.12) to generate corrected reads, which were further constructed using Celera Assembler version 8.3 and FALCON version 0.3.0 to yield optimal assemblies. The assemblies obtained were checked with Illumina sequences and, to improve the accuracy of the genome assemblies, GATK version 1.6-13 and SOAP tool packages (SOAP2, SOAPsnp, and SOAPindel) were used to make single-base corrections. Contig circle analysis was conducted by verifying overlap regions of >3,000 bp.

**TABLE 1:** General statistics of Illumina and PacBio sequencing runs

Sequencing platform	No. of reads	Length of reads (bp)	Sequencing coverage (×)	Amt of data (Mb)
Illumina	5,617,276	150	232	842
PacBio	258,584	7,579 (mean)	612	1,960

CBW1107 contains one circular chromosome with no plasmids. The CBW1107 genome is composed of 3,202,093 bp, 3,446 coding sequences, a GC content of 66.86%, and 54 noncoding RNAs. Open reading frame (ORF) prediction was performed on the CBW1107 genome using Glimmer version 3.02 (http://ccb.jhu.edu/software.shtml) with hidden Markov models. tRNA, rRNA, and small RNA (sRNA) recognition was performed using tRNAscan-SE version 1.3.1 (6), RNAmmer version 1.2, and the Rfam version 9.1 databases. The finding of rRNAs was performed by comparison with the rRNA database or prediction with RNAmmer software; tRNAscan was used to predict the areas of tRNAs and their secondary structures, and Infernal was used to compare sequences with the Rfam database and to obtain sRNAs. Seven databases were used for ORF function annotation, namely, Kyoto Encyclopedia of Genes and Genomes (KEGG) database version 81, Clusters of Orthologous Groups (COG) database version 2014-11-10, nonredundant protein database version 2017-10-10, Swiss-Prot database version 2017-07, Gene Ontology (GO) database release 2017-09-08, TrEMBL database release 2017-09, and EggNOG database version 4.5. To predict the biological meaning, the highest-quality alignment result was chosen for gene annotation.

The genome of CBW1107 contains genes associated with lipid membrane synthesis and desaturation, including genes that modify membrane fluidity. It also includes chaperone proteins to ensure proper protein folding. These genomic features suggest that this strain has the ability to adapt and to subsist under a wide range of environmental conditions. To the best of our knowledge, the genome sequence of *Synechococcus* sp. strain CBW1107 represents the first complete genome sequence for subalpine cluster II.

### Data availability.

The genome sequence of *Synechococcus* sp. strain CBW1107 was deposited in GenBank under the accession number CP064908, where PGAP annotation is also available. Reads were deposited in the Sequence Read Archive (SRA) under accession numbers SRR13195542 (for PacBio reads) and SRR13195543 (for Illumina reads) under BioProject accession number PRJNA657291 and BioSample number SAMN16790668.
